# Auricular Cartilage Preservation Through Abdominal Subcutaneous Implantation Following Traumatic Ear Amputation: A Case Report

**DOI:** 10.7759/cureus.112141

**Published:** 2026-07-06

**Authors:** Abdelouahid Taleuan, Adnane Lakhal, Mohammed ElAmine Squalli Houssaini, Abdellatif Oudidi

**Affiliations:** 1 Department of Otolaryngology - Head and Neck Surgery, Al Amir Sultan Hospital, Errachidia, MAR; 2 Department of Otolaryngology - Head and Neck Surgery, Centre Hospitalier Universitaire Hassan II, Fez, MAR

**Keywords:** auricular amputation, cartilage preservation, ear reconstruction, road traffic accident, staged reimplantation, subcutaneous implantation

## Abstract

Traumatic auricular amputation is a rare but highly disfiguring injury that poses significant reconstructive challenges. When microsurgical replantation is not feasible, subcutaneous implantation of the auricular cartilage framework, typically in the abdominal wall, is an established technique for cartilage preservation prior to delayed reconstruction. We report a case of successful auricular cartilage preservation and reimplantation following traumatic ear amputation sustained in a road traffic accident. A 22-year-old man presented with complete traumatic amputation of the left ear and an extensive retroauricular soft tissue defect following a road traffic accident. Microsurgical replantation was not feasible due to the extent of soft tissue damage. The auricular cartilage was harvested, cleaned, and implanted subcutaneously in the anterior abdominal wall for preservation. After three months, once the operative site had healed satisfactorily, the preserved cartilage framework was retrieved and reimplanted in the auricular region. The final aesthetic outcome was good, with acceptable morphology and adequate structural support. Subcutaneous abdominal implantation of auricular cartilage is a reliable and effective technique for cartilage preservation when primary replantation is not possible. This staged approach allows subsequent reconstruction with preserved autologous tissue, offering satisfactory functional and cosmetic results.

## Introduction

Traumatic amputation of the auricle is a rare but functionally and aesthetically significant injury, representing one of the most challenging scenarios in reconstructive surgery [1,2]. The auricle's complex three-dimensional architecture, combined with its limited soft tissue envelope and poor vascularity, makes replantation and reconstruction technically demanding [3].

When microsurgical replantation is feasible, it remains the gold standard, restoring native auricular anatomy and achieving optimal aesthetic results [4,5]. However, in cases complicated by extensive soft tissue loss, vascular damage, or absence of suitable recipient vessels, as frequently encountered in high-energy trauma, alternative reconstructive strategies must be employed [6]. The main reconstructive options in such cases include subcutaneous abdominal preservation of the native cartilage framework, construction of a costal cartilage framework, and alloplastic prostheses [7,8].

Subcutaneous abdominal implantation of the amputated auricular cartilage, first described by Baudet et al. in 1972, offers a biologically sound method of cartilage preservation that maintains chondrocyte viability through vascularization by the surrounding subcutaneous tissue [9,10]. This staged technique avoids the need for microsurgery and preserves the native cartilage architecture for subsequent reconstruction [11,12].

We report a case of complete traumatic auricular amputation managed by this staged approach, with a total reconstruction timeline of six months, and discuss our results in the context of the current literature [13].

## Case presentation

A 22-year-old man was admitted to the emergency department on Day 0 following a road traffic accident involving a motor vehicle. He presented with complete traumatic amputation of the left auricle and an extensive retroauricular soft tissue defect with significant tissue loss. The amputated ear specimen was recovered at the scene and brought to the hospital (Figure 1).

**Figure 1:**
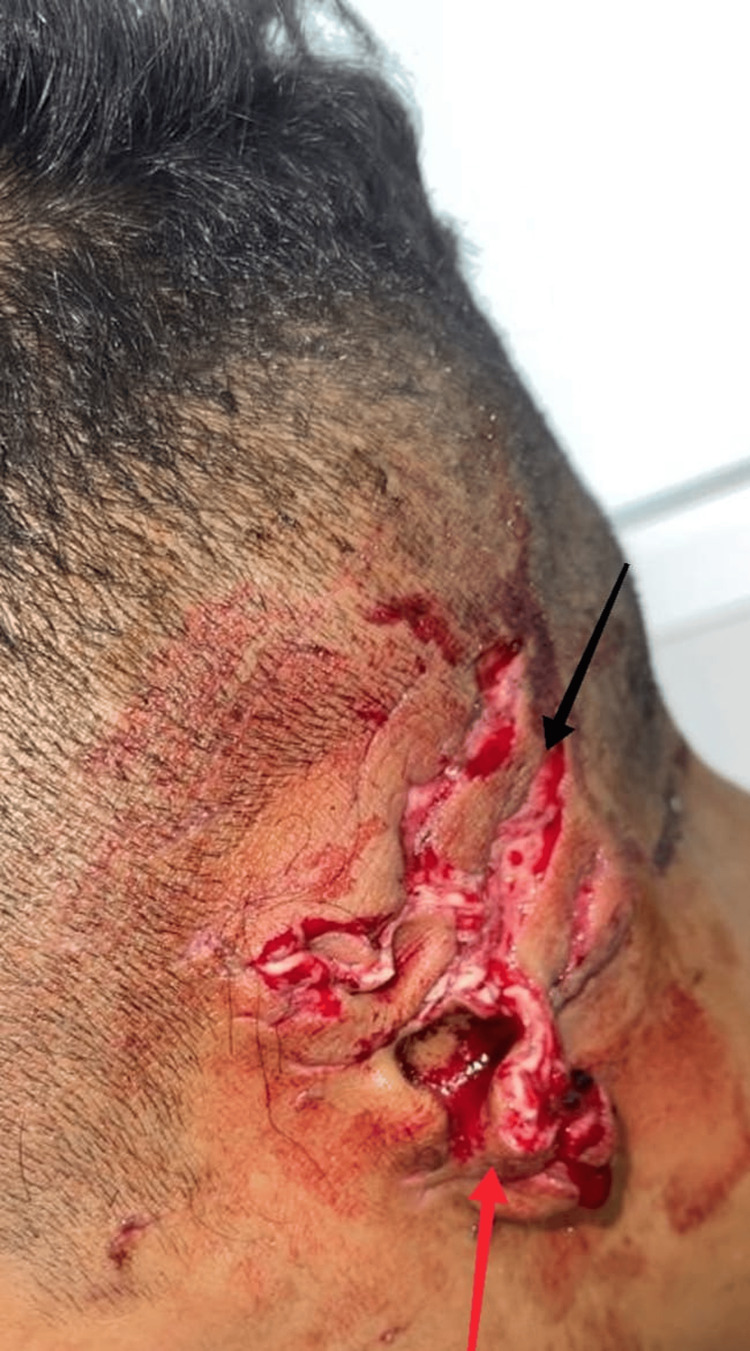
Initial presentation showing complete traumatic amputation of the left auricle following a road traffic accident. The black arrow indicates the retroauricular soft tissue defect. The red arrow indicates the amputation site with exposed cartilaginous remnants

On examination, the wound demonstrated extensive soft tissue damage involving the retroauricular region, with loss of overlying skin and subcutaneous tissue. The auricular cartilage framework was largely intact within the amputated specimen. General physical examination was otherwise unremarkable. No associated craniofacial or cervical injuries were identified.

Otoscopic examination of the ipsilateral external auditory canal and tympanic membrane was performed at initial presentation and revealed no structural abnormalities. The tympanic membrane appeared intact and translucent with a normal light reflex. Audiometric evaluation, including pure-tone audiometry, demonstrated normal hearing thresholds bilaterally, with no evidence of conductive or sensorineural hearing loss attributable to the traumatic event. These baseline audiological findings confirmed the absence of middle- or inner-ear involvement and were documented for comparison with postoperative assessments.

Surgical decision and cartilage harvesting

The patient underwent surgery on Day 1, within 24 hours of the initial trauma. Given the extent of the soft tissue avulsion and the absence of suitable recipient vessels for microsurgical anastomosis, primary replantation was deemed infeasible. A staged reconstructive approach was therefore planned, beginning with preservation of the auricular cartilage via subcutaneous abdominal implantation (Figure 2).

**Figure 2:**
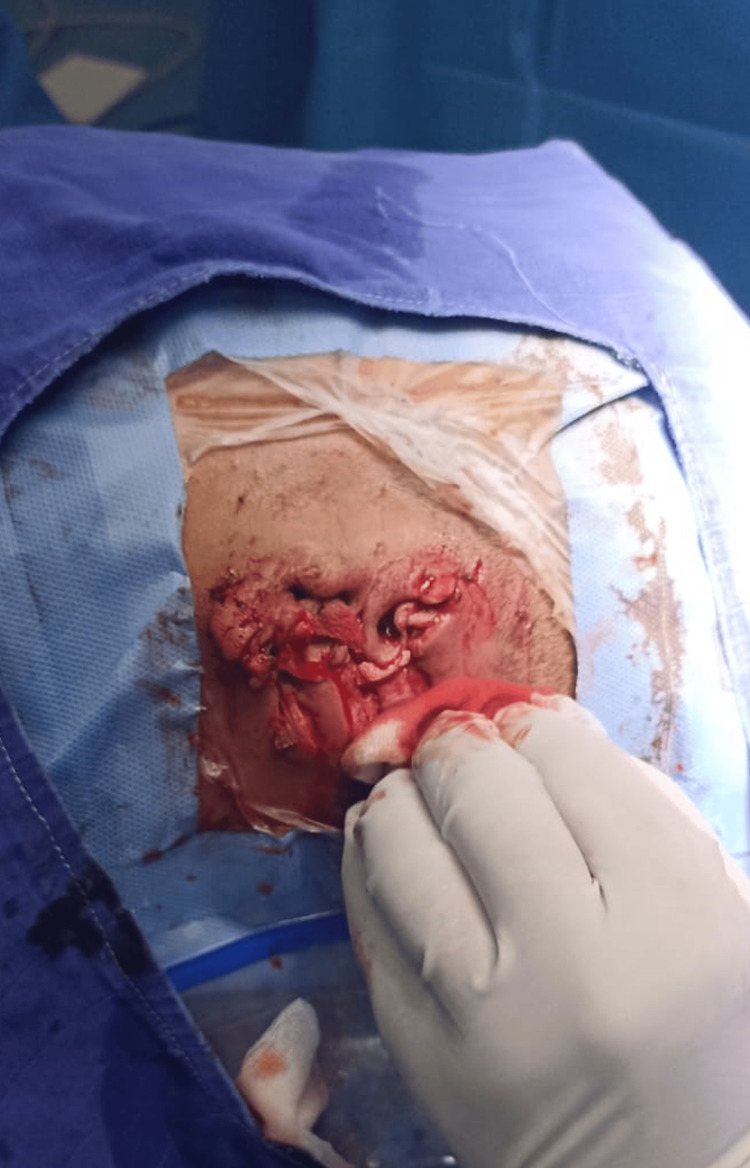
Intraoperative view of the amputated auricular specimen during debridement and preparation of the cartilaginous framework prior to abdominal implantation

The amputated auricle was thoroughly cleaned and debrided in the operating theater under aseptic conditions. The overlying skin was carefully removed from the auricular specimen, and the cartilaginous framework was isolated (Figure 3). The cartilage framework was inspected and found to be structurally intact, making it suitable for preservation.

**Figure 3:**
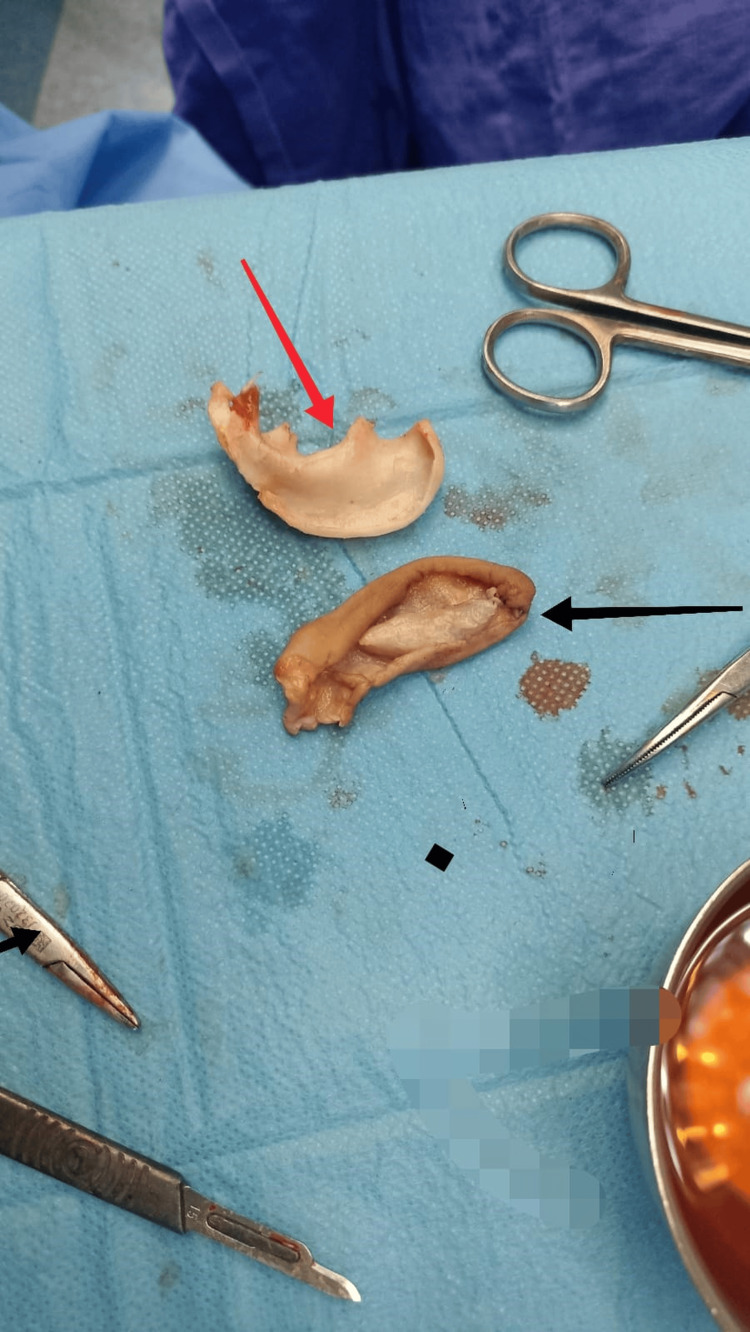
Harvested auricular cartilage framework on the instrument table after removal of overlying skin and soft tissue. The red arrow indicates the cartilage framework. The black arrow indicates the residual skin and soft tissue removed from the auricular specimen

Abdominal subcutaneous implantation

Under general anesthesia, the auricular cartilage framework was implanted subcutaneously in the anterior abdominal wall on Day 1 (Figure 4).

**Figure 4:**
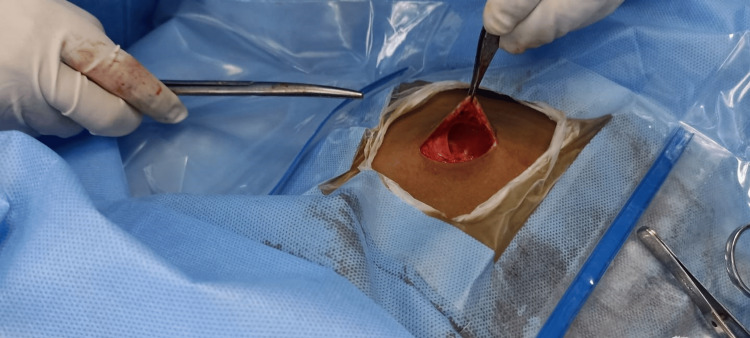
Intraoperative view of the anterior abdominal wall showing the subcutaneous pocket created by blunt dissection for implantation of the auricular cartilage framework

A small transverse incision was made in the anterior abdominal wall, and a subcutaneous pocket was carefully created by blunt dissection. The cartilage framework was positioned within the pocket in its anatomical orientation, and the wound was closed in layers over the implant (Figure 5).

**Figure 5:**
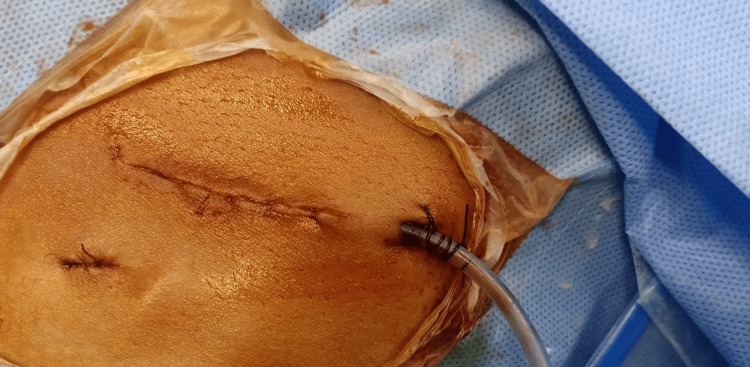
Immediate postoperative view of the abdominal implantation site after wound closure over the subcutaneously preserved auricular cartilage, with a suction drain in place to prevent hematoma formation

Postoperative course and wound management

The immediate postoperative period following abdominal implantation was uneventful. The suction drain was removed on Day 3, once drainage was minimal. Wound healing at the abdominal implantation site progressed satisfactorily throughout the three-month preservation period, with no signs of infection, cartilage protrusion, or wound breakdown (Figure 6).

**Figure 6:**
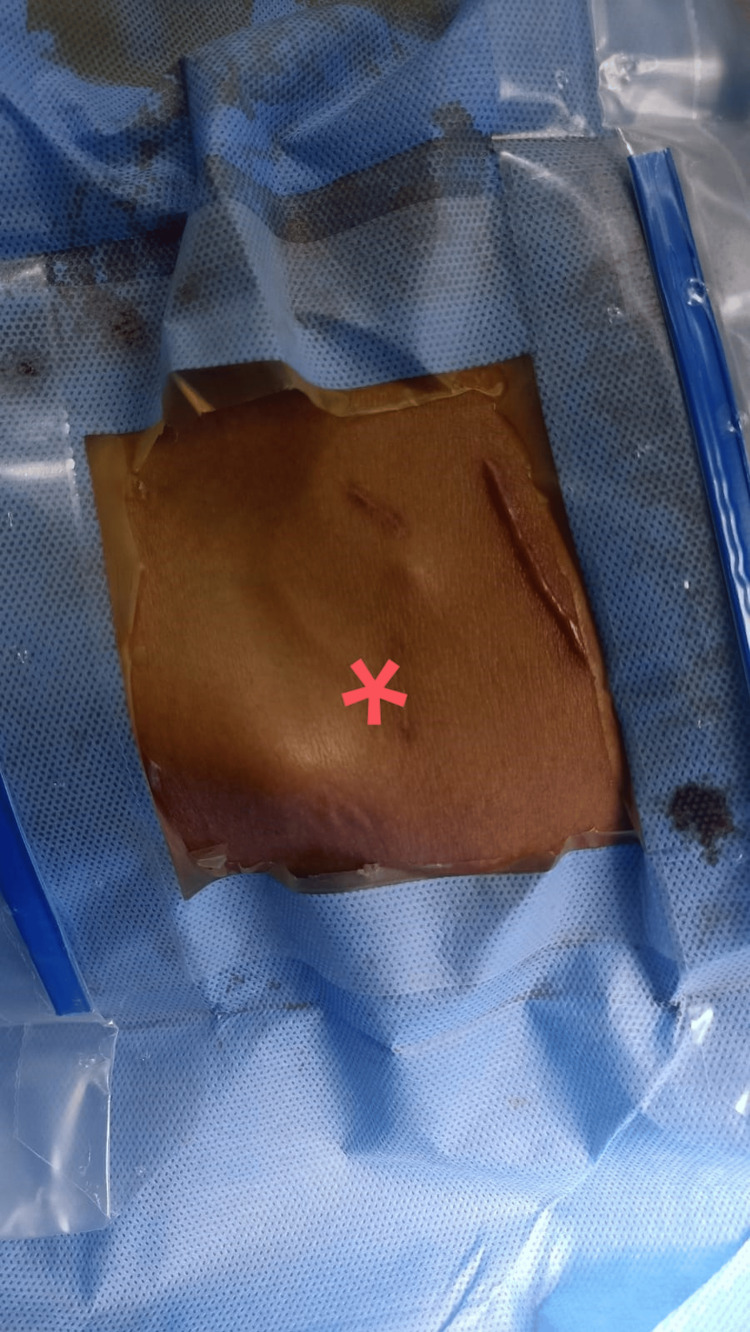
Healed abdominal implantation site at three months, demonstrating a well-closed wound with no evidence of infection, wound dehiscence, or subcutaneous cartilage protrusion. The red asterisk (*) indicates the healed subcutaneous implantation site

Simultaneously, the retroauricular defect was managed with appropriate wound care and local dressings, resulting in satisfactory soft tissue maturation at three months (Figure 7).

**Figure 7:**
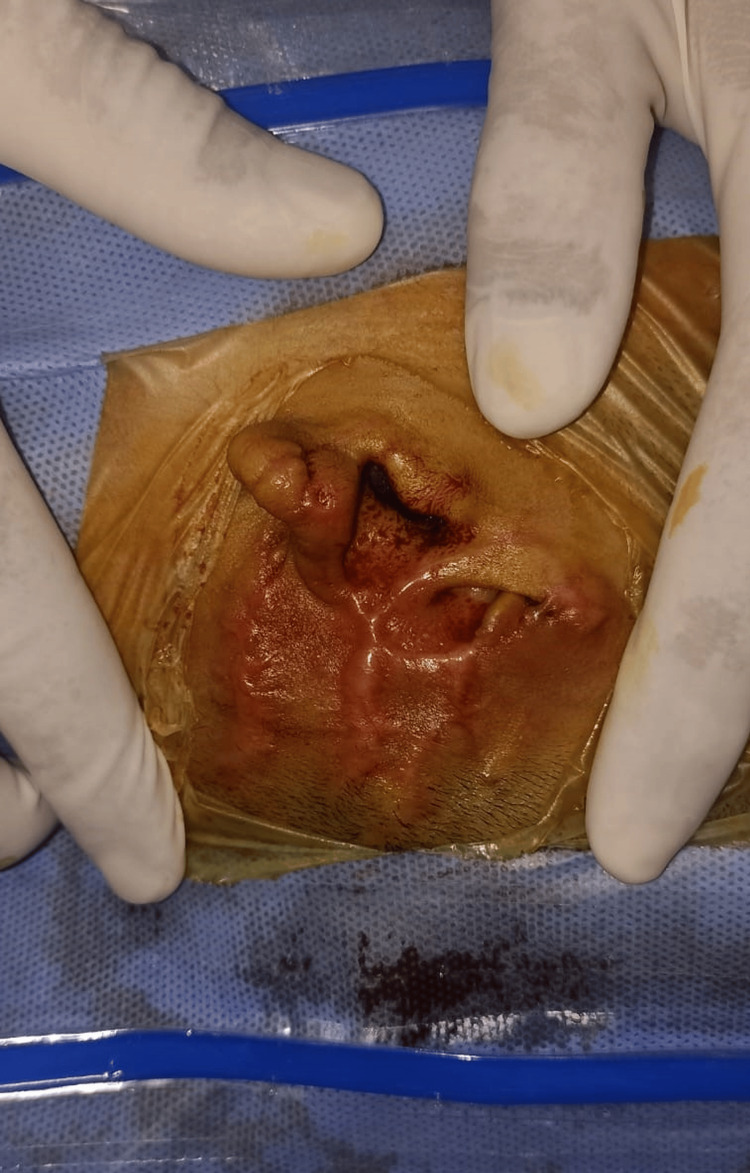
Auricular operative site at three months postoperatively, showing complete healing and satisfactory soft tissue maturation before definitive auricular reconstruction

Cartilage retrieval and staged reimplantation

Three months after the initial procedure, once the operative sites had fully healed and the patient was in satisfactory general condition, the preserved cartilage was retrieved from the abdominal subcutaneous pocket under general anesthesia (Figure 8).

**Figure 8:**
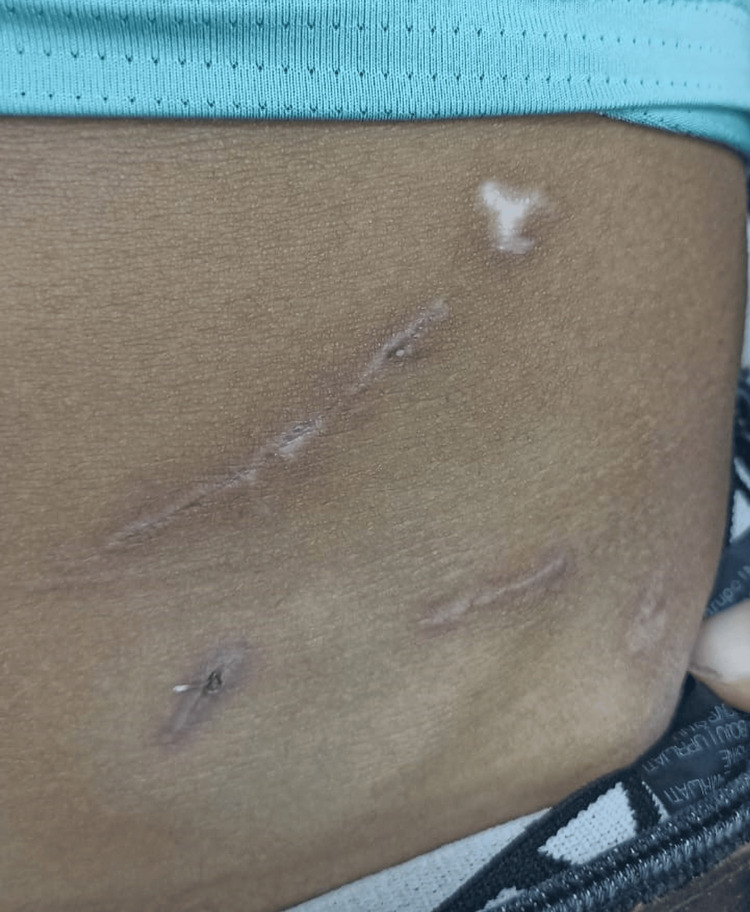
Appearance of the abdominal donor site at three months following cartilage retrieval, showing a discreet, well-healed linear scar with minimal aesthetic impact, an important advantage of this technique

Intraoperatively, the cartilage framework was well preserved, viable, and structurally intact, with no evidence of resorption, infection, or structural degradation, confirming the efficacy of the abdominal subcutaneous environment as a biological storage medium (Figure 9).

**Figure 9:**
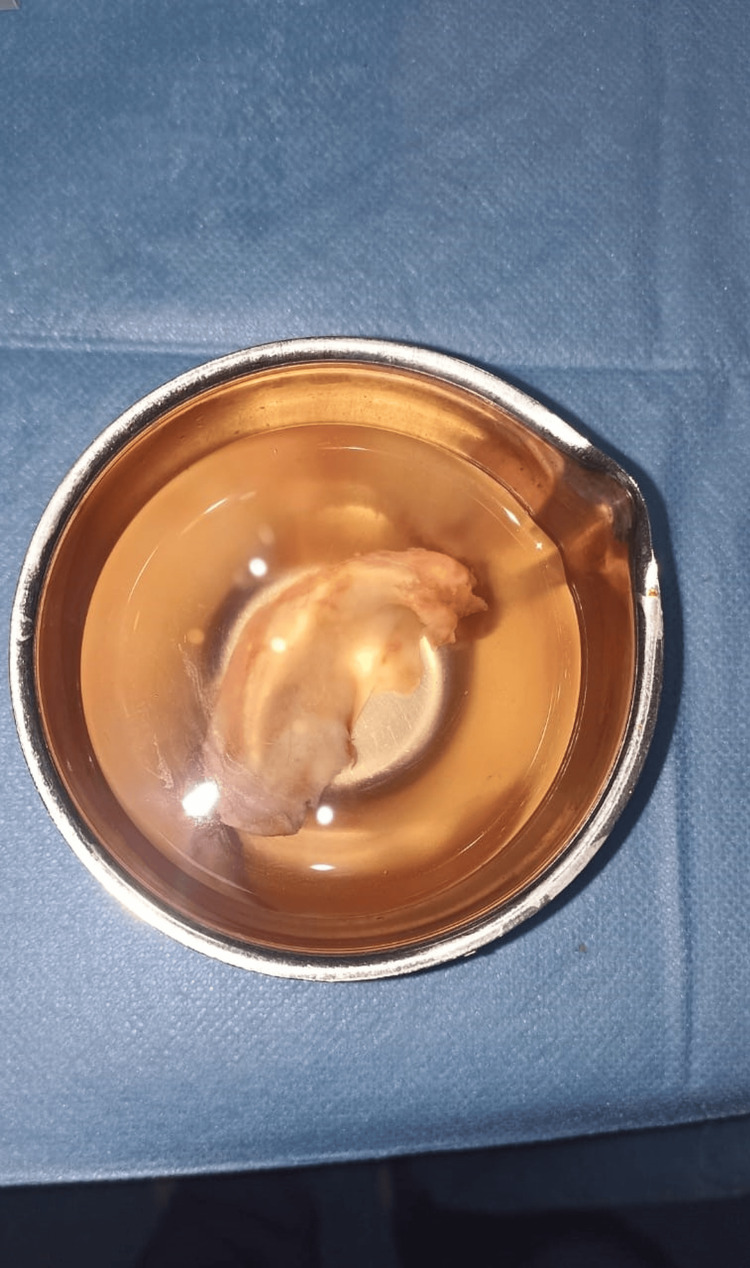
Retrieved auricular cartilage framework after three months of subcutaneous abdominal preservation, demonstrating intact helical and antihelical architecture with maintained cartilage viability and no evidence of structural resorption

The cartilage was then reimplanted in the left auricular region (Figure 10).

**Figure 10:**
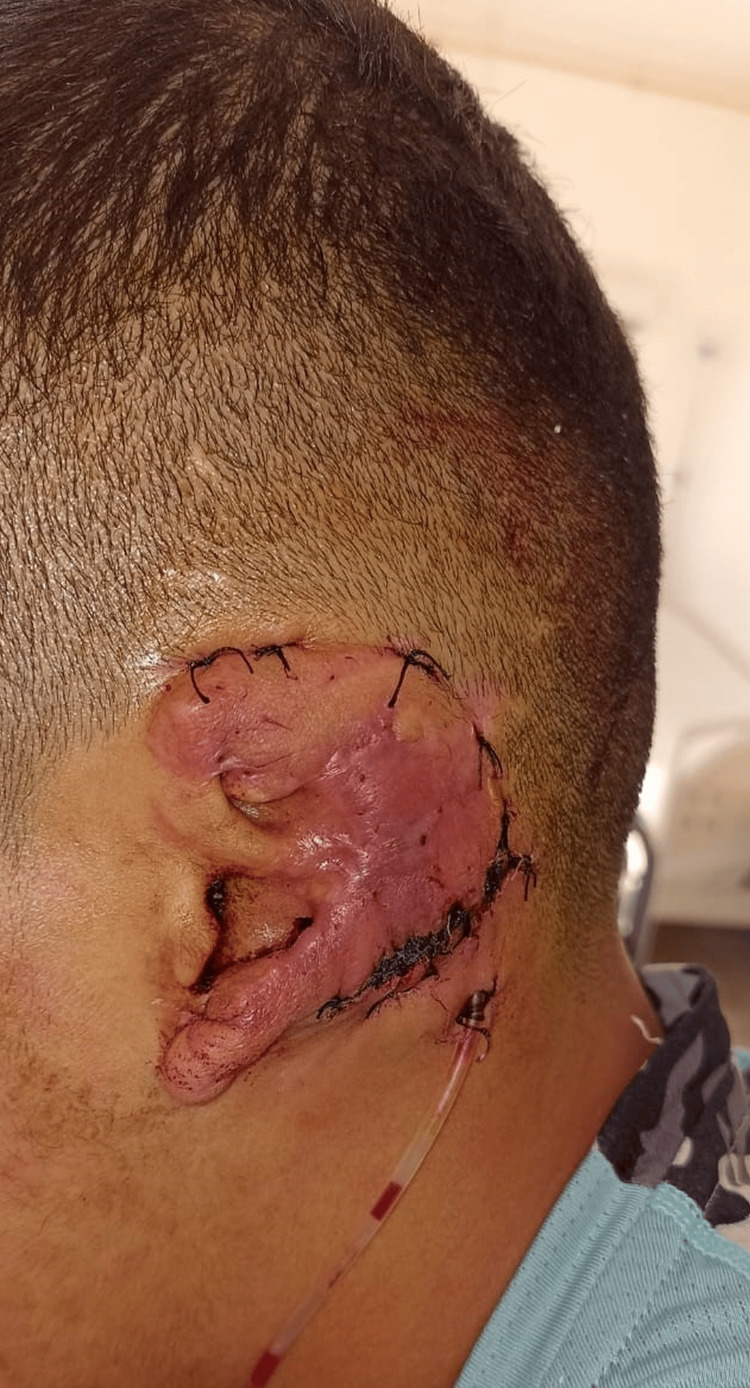
Early postoperative view of the left auricular region following management of the retroauricular soft tissue defect, with suction drain in place

The retroauricular wound bed was prepared by careful excision of scar tissue, and a well-vascularized subcutaneous pocket was created to receive the preserved cartilage framework. The auricular cartilage was positioned in its correct anatomical orientation and secured with absorbable sutures (Figure 11).

**Figure 11:**
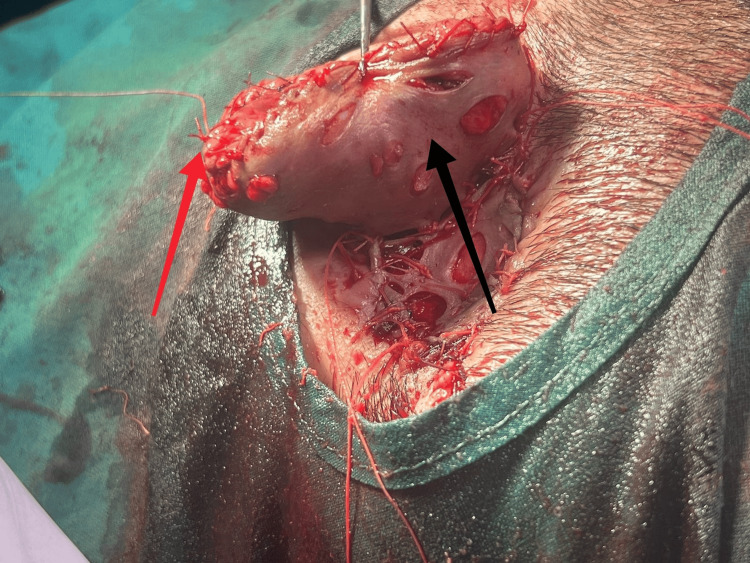
Intraoperative view during staged auricular reimplantation showing the cartilage framework repositioned in its anatomical location. The red arrow indicates the cartilage framework with sutures in place and vascularized soft tissue coverage. The black arrow indicates the skin grafting area, achieving complete coverage of the reconstructed auricle

Skin coverage was achieved using local advancement flaps and direct closure over the reconstructed framework (Figure 12).

**Figure 12:**
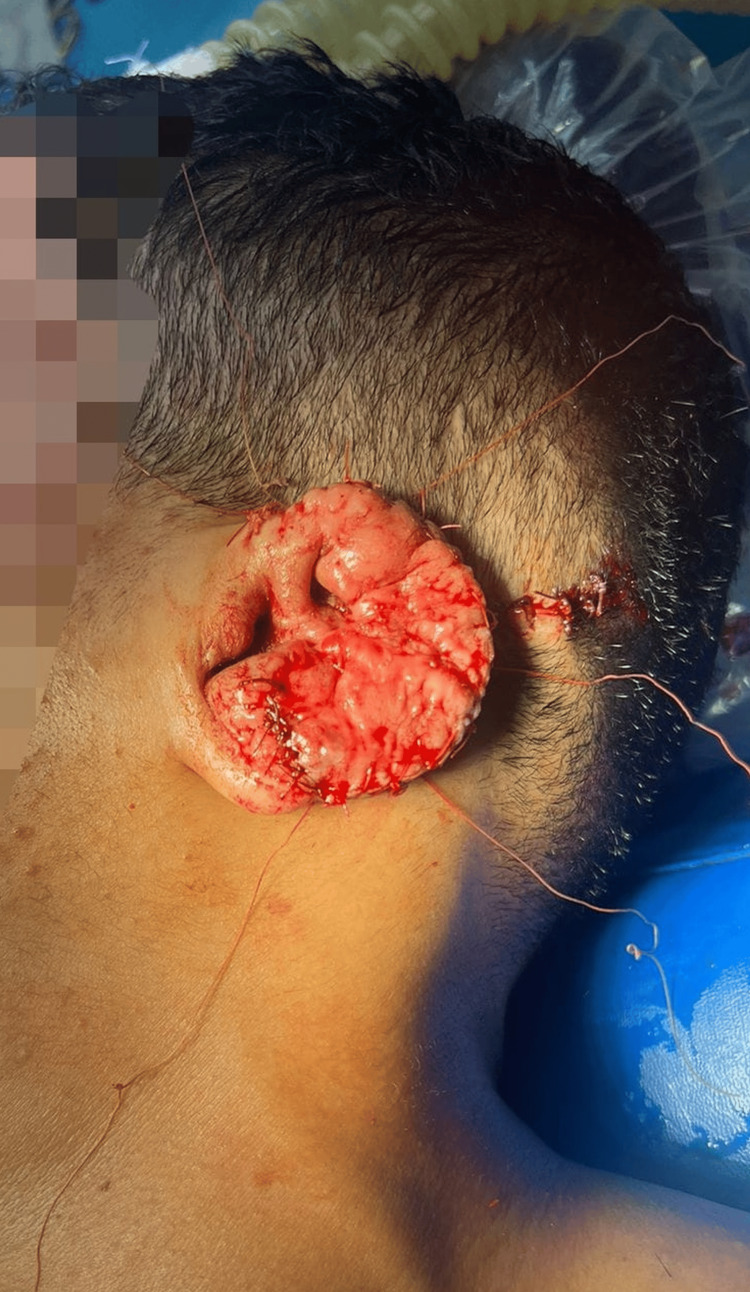
Intraoperative overview immediately following completion of auricular reimplantation, demonstrating satisfactory restoration of auricular projection, position, and overall morphology relative to the lateral face

Definitive reconstruction and final outcome

Three months after cartilage retrieval, definitive auricular reconstruction was completed, resulting in a total treatment period of six months from the initial trauma. The postoperative course following reimplantation was uneventful, with no wound complications, hematoma, or signs of cartilage extrusion. At the three-month postoperative follow-up, the patient demonstrated satisfactory wound healing and good integration of the reconstructed auricle. The overall aesthetic outcome was judged to be good, with acceptable auricular morphology, adequate three-dimensional projection, and natural positioning relative to the face (Figure 13).

**Figure 13:**
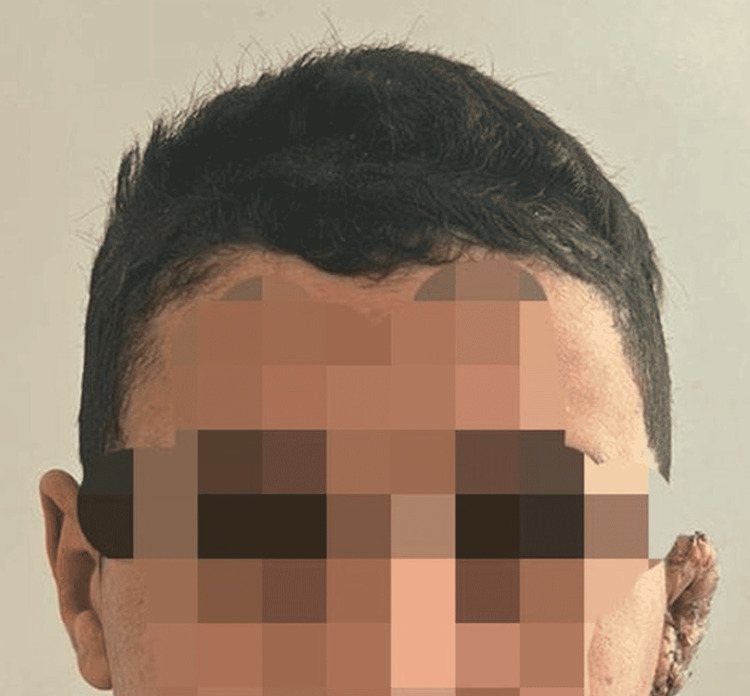
Final postoperative result at three months following staged auricular reimplantation. The reconstructed left ear demonstrates satisfactory morphology, adequate projection, and an acceptable aesthetic outcome. The patient is in good general condition and expressed satisfaction with the result

Mild distortion of the helical contour was noted, which is a well-recognized and acceptable sequela of this staged technique. The patient expressed satisfaction with the functional and cosmetic result.

Postoperative audiological assessment was performed at the three-month postoperative follow-up. Otoscopic examination confirmed intact tympanic membrane integrity bilaterally, with no evidence of canal obstruction, effusion, or structural change. Pure-tone audiometry demonstrated stable hearing thresholds relative to baseline, with no deterioration in auditory function attributable to either the traumatic event or the surgical procedures. These findings confirm the preservation of hearing function throughout the reconstructive course and strengthen the overall functional outcome of this staged auricular reconstruction technique.

## Discussion

Traumatic amputation of the auricle is a rare but surgically challenging injury that requires careful planning and a staged reconstructive approach [1,2]. The management strategy depends on several factors, including the viability of the amputated segment, the extent of soft tissue loss, and the availability of recipient vessels for microsurgical replantation [3].

Choice of reconstructive strategy

Microsurgical replantation remains the gold standard when technically feasible, offering the best aesthetic outcome by restoring the native auricular architecture [4,5]. However, in cases where the soft tissue envelope is severely damaged or suitable recipient vessels are absent, as in our patient, alternative strategies must be considered.

Costal cartilage reconstruction, first described by Tanzer and later refined by Nagata, involves carving a three-dimensional auricular framework from the patient's own rib cartilage [7]. While this technique offers excellent long-term results and avoids the risk of implant rejection, it requires significant surgical expertise, involves donor site morbidity at the chest wall, and necessitates multiple staged procedures over an extended period [13].

Alloplastic prostheses, including Medpor (porous polyethylene) and silicone frameworks, offer alternatives that avoid donor-site morbidity and reduce operative time [14]. However, they carry a higher risk of extrusion, infection, and implant exposure, particularly in the setting of traumatic injury where the soft tissue envelope may be compromised [15].

In our patient, subcutaneous abdominal preservation of the native auricular cartilage was selected as the optimal strategy, as it preserves the patient's own cartilage architecture, avoids donor site morbidity, maintains cartilage viability through vascularization from the surrounding subcutaneous tissue, and allows definitive reconstruction once the local soft tissues have adequately healed [8,9].

Abdominal subcutaneous preservation: technique and outcomes

The abdominal subcutaneous environment provides an ideal biological storage medium for auricular cartilage, offering adequate vascularization to maintain chondrocyte viability and structural integrity [16]. In our case, the cartilage was retrieved after three months and found to be completely intact with no evidence of resorption, consistent with previously published series reporting successful cartilage preservation over periods ranging from six weeks to six months [10-12].

The three-month preservation interval chosen in our case represents a well-established compromise between allowing adequate maturation of retroauricular soft tissues and minimizing the risk of progressive cartilage resorption with prolonged implantation [16]. The retroauricular wound was managed conservatively with local dressings during this interval, allowing complete soft tissue healing prior to definitive reconstruction.

Complications and risk mitigation

Potential complications of this staged technique include cartilage resorption, abdominal wound infection, hematoma formation, cartilage extrusion, and suboptimal aesthetic outcomes due to helical contour distortion [17]. In our patient, none of these major complications were observed.

To minimize complications, meticulous hemostasis, suction drainage for 48 hours, and careful pocket closure are essential technical steps [11]. Additionally, timing of cartilage retrieval should be guided by complete healing of both the abdominal and auricular sites, as premature retrieval increases the risk of wound complications and cartilage damage [16].

Comparison with published literature

Recent systematic reviews have confirmed that non-microsurgical techniques, including subcutaneous preservation, achieve acceptable outcomes in settings where microsurgical expertise is unavailable [18,19]. Arterial-only anastomosis associated with modified Baudet techniques has also demonstrated promising outcomes in selected cases of auricular replantation [20]. Contemporary reviews continue to support autologous cartilage preservation techniques as reliable alternatives when microsurgical replantation is not feasible [21,22]. Novel modifications of pocket preservation methods have recently been described with encouraging aesthetic and functional outcomes [23].

Our case adds to the growing body of evidence supporting subcutaneous abdominal preservation as a viable and reproducible technique for auricular cartilage salvage following traumatic amputation. The six-month total reconstruction timeline observed in our patient is consistent with previously reported series. The satisfactory aesthetic outcome achieved further supports the utility of this approach, particularly in resource-limited settings where microsurgical facilities may not be readily available.

## Conclusions

Subcutaneous abdominal implantation of the auricular cartilage framework is a reliable, reproducible, and effective technique for cartilage preservation following traumatic auricular amputation when microsurgical replantation is not feasible. The staged approach, acute cartilage preservation followed by delayed reimplantation, allows reconstruction using the patient’s own preserved auricular framework, with acceptable aesthetic outcomes and minimal donor site morbidity. Early recognition and prompt cartilage salvage at initial presentation are essential to the success of this technique. This case demonstrates that satisfactory auricular reconstruction can be achieved even in resource-limited settings. It reinforces the importance of awareness of this technique among otolaryngologists and reconstructive surgeons managing complex maxillofacial trauma.
